# History of Hypertension From Childhood and Fasting Blood Glucose Levels in Adulthood: The Bogalusa Study

**DOI:** 10.1111/1753-0407.70155

**Published:** 2025-10-01

**Authors:** Lingli Zhao, Jiahui Ouyang, Yewen Song, Hua Qu, Zhuye Gao

**Affiliations:** ^1^ Xiyuan Hospital, China Academy of Chinese Medical Sciences Beijing China; ^2^ National Clinical Research Center for Chinese Medicine Cardiology Beijing China

**Keywords:** blood pressure, body mass index, fasting blood glucose, regression analysis, vascular dysfunction

## Abstract

**Background:**

Fasting blood glucose (FBG) reflects cardiometabolic health, but the long‐term effects of childhood hypertension (HTN) on adult FBG are unclear.

**Methods:**

Using data from the Bogalusa Heart Study, we examined the link between childhood HTN and FBG in early adulthood, adjusting for race, BMI, pulse rate, and blood pressure.

**Results:**

Individuals with childhood HTN had higher FBG in early adulthood (mean difference 8.96 mg/dL) and a 4.16‐fold higher risk of high FBG (≥ 126 mg/dL). The effect was stronger in African Americans, those with higher pulse rate, overweight individuals, or those with low diastolic BP.

**Conclusions:**

Childhood HTN is linked to elevated FBG in early adulthood. Early management of hypertensive children, especially those at metabolic risk, may help prevent diabetes and cardiovascular disease later.

## Graphical abstract

Childhood hypertension was associated with a fourfold higher risk of elevated fasting blood glucose in adulthood, independent of multiple confounders.

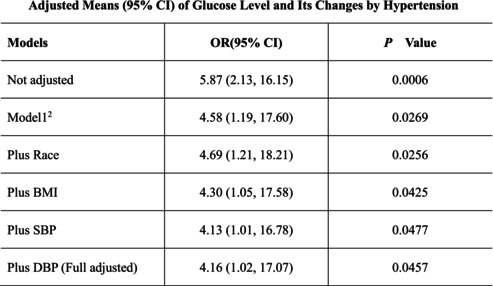

**Summary**

- Our analysis indicates a potential relationship between a history of childhood hypertension and elevated blood glucose levels in adulthood.
- A retrospective study of participants in the Bogalusa Heart Study suggests that children with hypertension are at increased risk of abnormal blood glucose in adulthood.

To the Editor,

Diabetes and prediabetes are well‐known risk factors for cardiovascular disease, associated with increased mortality [1]. Elevated blood glucose levels are linked to cardiovascular health and higher mortality [2, 3, 4]. Effective glycemic control can significantly reduce the incidence of diseases such as myocardial infarction [5, 6]. The prevalence of hypertension (HTN) among children and adolescents declined from 6.6% in 1999 to 2.5% in 2014. But recent increases, especially due to obesity, have been observed [7, 8]. Early‐onset HTN exerts long‐term and adverse effects on cardiovascular health in adulthood. This study used data from the Bogalusa Heart Study to evaluate the relationship between a history of HTN during childhood and adolescence and fasting blood glucose (FBG) levels in adulthood.

## 1 Methods

### 1.1 Study Cohort

**FIGURE 1:**
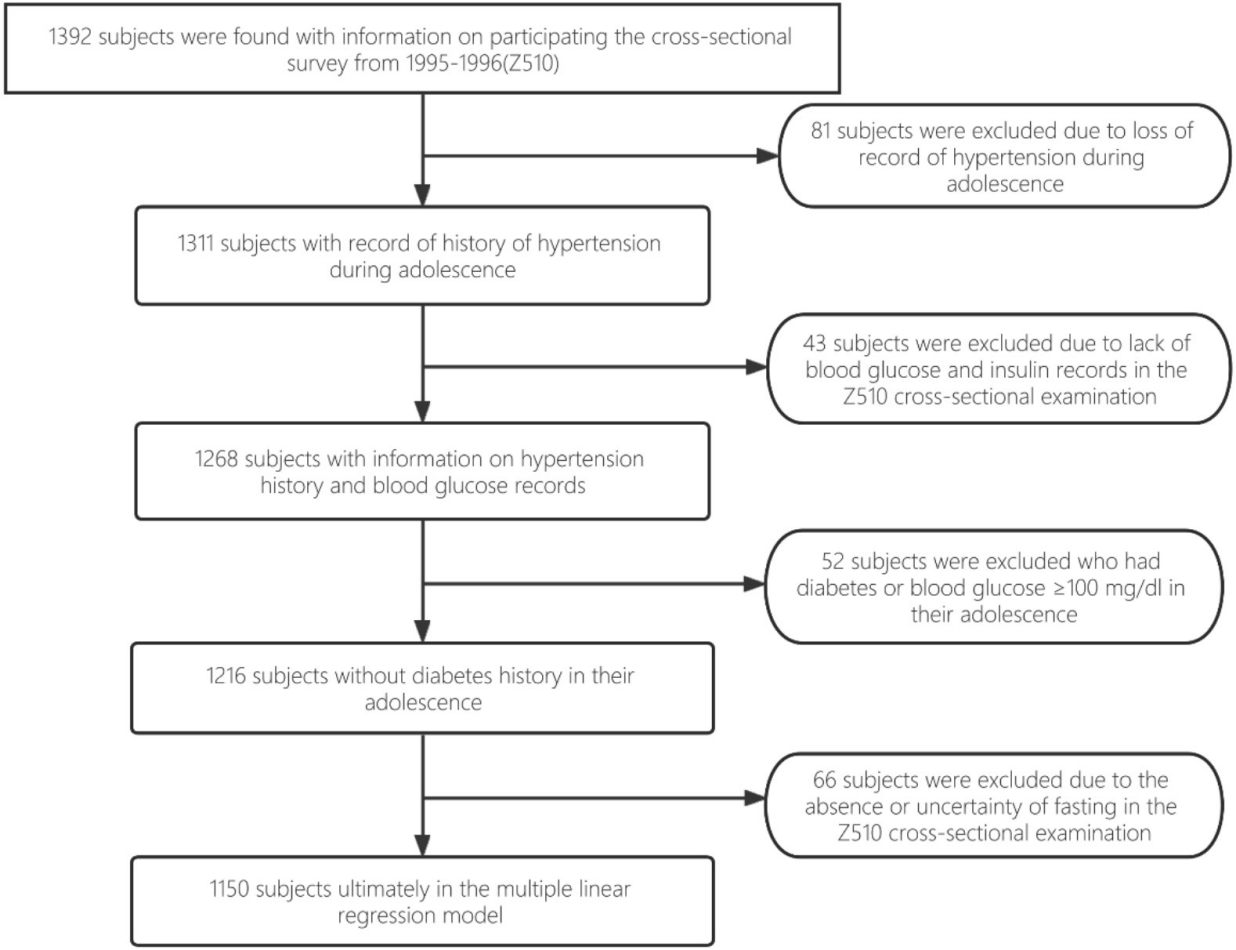
Flowchart of the study.

The Bogalusa Heart Study aimed to investigate cardiovascular risk factors in the community. Between 1973 and 1996, eight cross‐sectional surveys were conducted, including seven on children (ages 5–17) and one in adulthood. The longitudinal cohort included participants from at least one childhood survey and the adulthood survey (Figure 1).

### 1.2 General Examination

Participants were instructed to fast for at least 8 h before the examination. The history of diseases and medication was obtained through a questionnaire administered by the Bogalusa Heart Study staff in a quiet and confidential setting. Data on the history of childhood HTN and smoking and alcohol consumption in adulthood were recorded.

Blood pressure (BP) was measured on the right arm using a mercury sphygmomanometer, with the average of six measurements recorded. Due to the unreliability of a single BP measurement in childhood and adolescence, a guardian‐reported history of HTN was preferred. If unavailable, the participant's measured BP was used. BP was assessed according to the Fourth Report on Hypertension Diagnosis, Evaluation, and Treatment by the American Heart Association, with HTN defined as BP ≥ 95th percentile for age, gender, and height in prepubertal children (1–13 years) [9]. Participants were considered hypertensive if at least one high BP reading was documented across multiple examinations.

### 1.3 Statistical Analysis

Participants were divided into normal and elevated groups based on the diagnostic criteria for diabetes mellitus issued by the American Diabetes Association (blood glucose < 100 mg/dL and blood glucose ≥ 100 mg/dL) [10]. Fixed effects such as gender, age, race, insulin level, systolic and diastolic blood pressure (SBP and DBP), heart rate, lipid level, and body mass index (BMI) were adjusted. *β*‐values of < 0.05 were considered statistically significant.

## 2 Results

In the final cohort of 1150 participants, 39.82% were male, and 60.18% were female. 70.70% were Caucasian Americans, and 29.30% were African Americans. Among them, 3% had a history of childhood HTN. Participants with childhood HTN had a higher BMI (35.0 kg/m^2^ versus 27.3 kg/m^2^, *p* < 0.001) and BP (120.8 mmHg versus 110.9 mmHg SBP and 81.3 mmHg versus 73.7 mmHg DBP, *p* < 0.001) compared with those without a history of childhood HTN. (Table 1).

**TABLE 1:** Characteristics of study chorts by hypertension.

Variables	Without HBP (*n* = 1117)	With HBP (*n* = 33)	*p* ^a^
Age, years	29.3 ± 5.1	29.2 ± 5.1	0.922
Gender			0.959
Male, *n* (%)	445 (39.8%)	13 (39.4%)	
Famale, *n* (%)	672 (60.2%)	20 (60.6%)	
Race			< 0.001
Caucasian American, *n* (%)	800 (71.6%)	13 (39.4%)	
African American, *n* (%)	317 (28.4%)	20 (60.6%)	
BMI, kg/m^2^	27.3 ± 6.8	35.0 ± 9.5	< 0.001
Pluse, beats per min	71.7 ± 10.2	68.8 ± 14.4	0.696
HDL‐C, mg/dL	49.2 ± 13.7	47.9 ± 12.4	0.686
LDL‐C, mg/dL	120.9 ± 33.8	127.9 ± 25.3	0.124
TC, mg/dL	189.8 ± 38.6	194.3 ± 26.7	0.237
SBP, mmHg	110.9 ± 11.1	120.8 ± 13.4	< 0.001
DBP, mmHg	73.7 ± 8.8	81.3 ± 10.2	< 0.001
Smoking (%)	96.31	3.69	0.545
Alchohol drinking (%)	96.26	3.74	0.103

^a^ Two groups' characteristics were compared using the Kruskal–Wallis test or Fisher test for continuous variables and *χ* ^2^ test for categorical variables.

Logistic regression was used to assess the relationship between childhood HTN and elevated adulthood FBG. After full adjustment, participants with HTN in childhood or adolescence were 4.16 times more likely to have elevated FBG in adulthood compared with participants with no history of HTN (OR = 4.16, [95% CI: 1.02 to 17.07] *p* = 0.0457) (Table 2). Further adjustments did not significantly alter this correlation.

**TABLE 2:** Adjusted means (95% CI) of glucose level and its changes by hypertension.

Models	OR (95% CI)	*p*
Not adjusted	5.87 (2.13, 16.15)	0.0006
Model 1^a^	4.58 (1.19, 17.60)	0.0269
Plus Race	4.69 (1.21, 18.21)	0.0256
Plus BMI	4.30 (1.05, 17.58)	0.0425
Plus SBP	4.13 (1.01, 16.78)	0.0477
Plus DBP (Full adjusted)	4.16 (1.02, 17.07)	0.0457

^a^ Model 1 used a multiple linear regression model in participants with hypertension (*n* = 33) or without hypertesion (*n* = 1117), controlled for age (years), gender (male/female), LDL‐C (mg/dL), HDL‐C (mg/dL), TC (mg/dL), pluse (beats per min), subscapular skin fold (mm), smoking status (current smokers/noncurrent smokers), alchohol drinking status (current drinkers/noncurrent drinkers), use of antihypertensive medicine (yes/no), history of asthma (yes/no), history of liver disease (yes/no), history of coronary heart disease (yes/no), history of left ventricular hypertrophy (yes/no), history of serious diseases (yes/no) as fixed effects.

## 3 Comment

Numerous cohort studies and cross‐sectional investigations have shown that hyperglycemia is an independent risk factor for HTN across age groups and genders, contributing to elevated BP through endothelial damage, sodium retention, and sympathetic overactivity [11, 12]. HTN exacerbates insulin resistance, creating a vicious cycle. A study found significant blood glucose differences between hypertensive and non‐hypertensive college students [13]. A longitudinal study of Caucasian men showed BP levels up to 25 years ago predicting diabetes risk [14]. Our study is the first to analyze the relationship between childhood HTN and adult blood glucose levels.

The mechanisms linking elevated BP lead to high blood glucose are complex, involving hemodynamic changes, endothelial damage, neurohumoral‐immune system dysregulation, and antihypertensive drug effect. HTN causes vascular dysfunction, including endothelial dysfunction, chronic inflammation, and structural remodeling [15, 16]. Molecular pathways like the renin‐angiotensin‐aldosterone system, endothelin‐1, oxidative stress, and complement system contribute to HTN development [17, 18]. Chronic inflammation inhibits insulin signaling, exacerbates oxidative stress, and increases vascular resistance, leading to insulin resistance [19, 20]. Sympathetic dysregulation further weakens insulin signaling. Different antihypertensive drugs also impact blood glucose, with ACE inhibitors and ARBs reducing diabetes risk, while thiazides and β‐blockers increase it [21, 22, 23].

This study has several shortcomings that need to be addressed. First, the history of HTN was obtained through questionnaires, which might contain incorrect answers, and the BP measurement method in a cross‐sectional study did not fulfill the criteria for diagnosing HTN. Second, the sample size was insufficient, and only 33 patients with HTN in childhood or adolescence were finally included, which could affect the accuracy of the statistics. In the future, we will conduct research with larger sample sizes.

## Conflicts of Interest

The authors declare no conflicts of interest.
